# Drug-Eluting Stent Fracture within 6 Days after Stent Implantation

**Published:** 2011-02-01

**Authors:** J Zamany, Y Mahmoody, M A Ostavan

**Affiliations:** 1 Cardiovascular Research Center, Faghihi Hospital, Shiraz University of Medical Sciences, Shiraz, Iran

**Keywords:** Drug-eluting stent, Fracture, Implantation

## Abstract

A 63-year-old woman with typical chest pain referred to our center. Her risk factors were hypertension (HTN), hyperlipidemia (HLP), diabetic mellitus (DM), and smoking. Coronary angiography (CAG) revealed a tubular lesion (95%) in proximal part, and a diffuse lesion (95%) in the mid part of left anterior descending (LAD) with a good distal flow. Stenting of proximal and mid part lesion was done for her with 2 drug-eluting stent (paclitaxel-eluting coronary stent system, EURoCOR) with success. After 6 days, due to a typical chest pain without response to medications, the coronary angiography was performed showing a definite fracture of stent which was implanted in the mid part of left anterior descending artery (LAD) 6 days before admission.

## Introduction

Stent fracture is one of the possible causes of rest enosis after drug eluting stent (DES) implantation. Stent fracture is likely affected by mechanical stresses provoked by rigid structures and hinge points; also stent fracture might be associated with the high incidence of target lesion revascularization. Stent fractures have been reported as rare complications. In one study, the incidence of stent fracture was 3.2%[1] and in another was 2.6%.[2]

Stent fracture is known to occur in several conditions including in terms of vessel, fractures of stent are subject to develop in the area of increased rigidity (for example, stent fracture is found in the point of the maximal vessel curvature, overlapping site of stents, and tortuous vessels or calcified lesions) and from the point of procedure, long stent implantation and larger balloon at high pressure tend to fracture the stent.[3]

## Case report

The patient was a 63-year-old woman with typical chest pain. she suffered from risk factors of hypertension (HTN), hyperlipidemia (HLP), diabetic mellitus (DM), and smoking. She did not have any abnormality in her physical examination. The biochemical test results, including cardiac enzymes were all within normal limits. Her baseline electrocardiogram was a normal sinus rhythm with left bundle branch block (LBBB). Transthoracic echocardiography showed a 40% ejection fraction, left ventricular dilatation with hypokinesia in the lateral walls and dyskinesia of septal and apical walls.

**Fig. 1:**
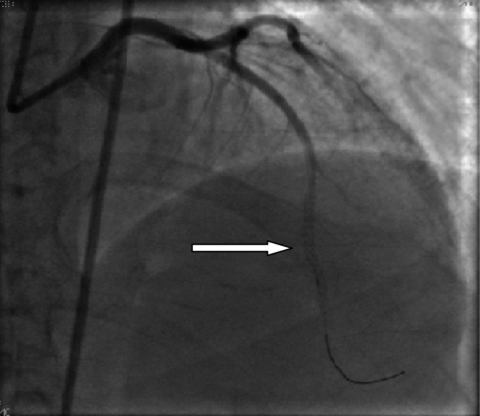
Coronary angiography after stenting shows a good deposition of stent

She underwent Coronary angiography (CAG) that revealed a tubular lesion (95%) in the proximal left anterior descending artery (LAD), and a diffuse lesion (95%) in mid part of LAD with a good distal flow. Stenting of proximal lesion with a 3 mm x 19 mm, paclitaxel-eluting coronary stent system (EURoCOR), and mid part of lesion with a 2.5 mm x 32 mm paclitaxel-eluting coronary stent system (EURoCOR) was carried out for her with success. After stenting, coronary angiography showed good deposition of stent with a good distal flow (TIMI3) (Figure 1). Afte 6 days, the patient referred with a typical chest pain without any response to medication.

**Fig. 2:**
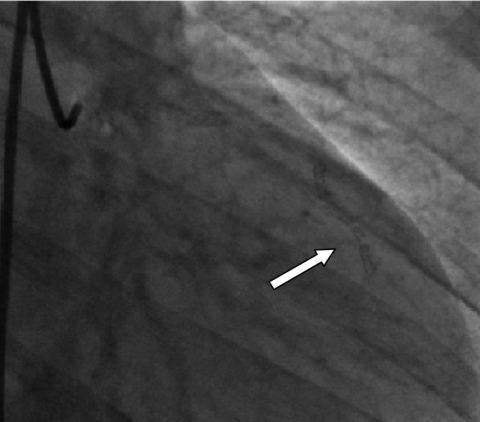
Coronary angiography 6 days after stent implantation shows the stent fracture (arrow)

Coronary angiography was done for her and showed an optimal extent expansion without any significant residual stenosis. However, we detected the definite fracture of stent, which was implanted in the mid part of LAD 6 days before her admission (Figure 2).

## Discussion

As drug-eluting stents greatly reduce the risk of in-stent rest enosis (ISR), it has been used extensively and may be used much more in the future.[3] Stent fractures were shown as rare complications and several cases of drug-eluting stent (DES) fracture have been reported.[4][5][6] They are germane to long coronary stents that became more prevalent with the advent of drug-eluting stents curbing the fear of long in-stent rest enosis. In one study the significant multivariate predictors of stent fracture were the stent length and the right coronary artery (RCA) location. The length of gap between the fractured segments and the time of stent fracture may contribute the ISR. Stent fractures are found in point of the maximal vessel curvature, overlapping site of stents, tortuous vessels, calcified lesions, long stent implantation and larger balloon at high pressure.[3] Stent fractures were previously reported several months after the follow-up of coronary angiographies, and the exact time when the stent fracture occurred was not tracked, but recently it has been reported until 2 days after Syfer eluting stent(SES) depvelopment.[3] We report a case with stent fracture in mid part of LAD within 6 days after paclitaxel-eluting coronary stent deployment.
